# TaqI, FokI, and ApaI Polymorphisms in the Vitamin D Receptor in Behçet's Disease in Turkish Population

**DOI:** 10.1155/2016/7475080

**Published:** 2016-09-01

**Authors:** Gaye Erten, Muhammed Kalkan, Sema Bilgiç Gazioğlu, Nilgun Akdeniz, Elif Ozkok, Burcak Vural

**Affiliations:** ^1^Department of Immunology, Aziz Sancar Institute of Experimental Medicine, Istanbul University, Istanbul, Turkey; ^2^Department of Genetics, Aziz Sancar Institute of Experimental Medicine, Istanbul University, Istanbul, Turkey; ^3^Department of Neuroscience, Aziz Sancar Institute of Experimental Medicine, Istanbul University, Istanbul, Turkey

## Abstract

*Objectives*. In our study we aimed to determine VDR gene polymorphisms in patients with Behçet's disease (BD) and neuro-Behçet's disease (NBD) in Turkish population.* Methods.* PBL obtained from 37 patients with BD, 21 patients with NB, and 30 healthy controls were investigated. Genomic DNA was extracted from whole blood using the QIAamp Blood Kit. VDR ApaI (rs7975232), VDR FokI (rs2228570), and VDR TaqI (rs731236) genotyping was performed by real-time polymerase chain reaction with SimpleProbe melting-curve analysis.* Results.* The allelic and genotype distributions of FokI and TaqI polymorphisms were not different among Behçet's disease, neuro-Behçet's disease, and control subjects in Turkish population (*p* > 0.05). Only the frequency of ApaI A allele in control is higher than that in BD (60% versus 38.5%), and the *p* value is 0.014, but the power is not enough to conclude that ApaI A allele is protective in BD in our study. Taken together, we found no significant differences between the BD, NBD, and control groups regarding the distribution of ApaI, TaqI, and FokI genotype and alleles frequencies.* Conclusions.* Future studies with larger patients' numbers may show differences between VDR polymorphisms and Behçet's disease.

## 1. Introduction

Behçet's disease (BD) is an autoinflammatory vasculitis described by a Turkish dermatologist, Behçet [1]. BD, also called Silk Road disease, is characterized by recurrent oral aphthous ulcers, genital ulcers, and uveitis [2]. Lately, BD has been classified as a mixed-pattern disease (in between monogenic autoinflammatory and autoimmune disorders) because of the human leukocyte antigen- (HLA-) B^*∗*^51 association [3].

Central nervous system (CNS) involvement, or neuro-Behçet's disease (NBD), develops in 5–10% of BD patients and generally affects the brain parenchyma and less frequently the brain vessels and meninges [4]. The cerebral parenchymal lesions are mainly composed of mononuclear and neutrophilic infiltrates [5]. In addition to genetic and environmental factors, vitamin D is known to play important role in the pathogenesis of Behçet's disease via immunologic mechanisms like T cell subsets, Treg cells, and intracytoplasmic cytokines expression [6]. Lower serum vitamin D levels were detected in patients with active BD [6, 7]. Vitamin D is primarily produced by ultraviolet irradiation of 7-dehydrocholesterol, but this primary form is metabolically inactive and has to be transformed to 25-hydroxyvitamin D3 in the liver followed by the formation of 1*α*,25-dihydroxyvitamin D3 in kidneys [8]. From an immunologic viewpoint, vitamin D is known to function by binding to its receptors (VDR) on immune cells like T lymphocytes and antigen presenting cells. Vitamin D suppresses B lymphocyte proliferation, differentiation, and antibody secretion [9]. It also has a negative effect on T lymphocyte proliferation but induces regulatory T cell production [10, 11]. The inhibitory effect of VitD3 on the Th17 and Th1 response was shown to be mediated via T cells and dendritic cells in patients with BD [12]. Vitamin D is implicated to affect endothelial functions, and replacement of vitamin D has been shown to have favorable effects on endothelial function [13]. Another study performed in 41 patients with BD suggested that the inflammation in BD was triggered through TLR2 and TLR4 and that vitamin D might be used as a therapeutic option for modulation of TLR2 and TLR4 expression of monocytes in BD [14].

The gene encoding VDR is located on chromosome 12q [15]. FokI (rs10735810), BsmI (rs1544410), TaqI (rs731236), and ApaI (rs7975232) polymorphisms are shown to play possible role in many diseases like cancer, infections, autoimmunity, allergy, and inflammation [16–20]. In our study, we aimed to determine VDR gene polymorphisms (FokI (rs2228570) (rs10735810), TaqI (rs731236), and ApaI (rs7975232)) in patients with BD and NBD in Turkish population.

## 2. Materials and Methods

### 2.1. Patients and Controls

PBL obtained from 37 patients with BD attending the Division of Rheumatology of Istanbul Faculty of Medicine and 21 patients with NB attending the Division of Rheumatology of Istanbul Faculty of Medicine, Istanbul University, were included in the study.

All patients fulfilled the diagnostic criteria of the International Study Group for BD [21]. Thirty controls DNA were provided by healthy blood donors with no known medical problems employed in the same hospital.

Patients gave informed consents, according to the principles of the Helsinki Declaration. The protocol used in the study was approved by the Local Ethical Committee of the Istanbul Medical Faculty, Istanbul University.

### 2.2. DNA Isolation and Genotyping

Genomic DNA was extracted from whole blood using the QIAamp Blood Kit (Qiagen, Inc.). VDR ApaI (rs7975232), VDR FokI (rs2228570), and VDR TaqI (rs731236) genotyping was performed by real-time polymerase chain reaction (RT-PCR, LightCycler, Roche) with SimpleProbe (TIB MOLBIOL) melting-curve analysis.

### 2.3. Statistical Analysis

Statistical analyses were performed using SPSS 21 (SPSS Inc., USA). The frequency of alleles and genotypes of VDR gene ApaI, FokI, and TaqI polymorphisms were analyzed by chi-square test. Hardy–Weinberg equilibrium (HWE) was determined by goodness-of-fit test. Differences were considered statistically significant at a *p* value <0.05.

## 3. Results

We studied the FokI, ApaI, and TaqI polymorphisms in 37 patients with BD, 21 patients with NBD, and 30 healthy controls. The Hardy–Weinberg principle was met in all groups.

Table 1 summarizes the distribution of genotype and alleles frequencies of VDR SNPs in patients with BD and control subjects.  Table 2 shows the distribution of genotype and alleles frequencies of VDR SNPs in patients with NBD and control subjects, and Table 3 reports the distribution of genotype and alleles frequencies of VDR SNPs in all patients (BD and NBD) and control subjects.

The distribution of ApaI genotypes was 33.3% for AC, 43.3% for AA, and 23.4% for CC in controls; 42.9% for AC, 17.1% for AA, and 40% for CC in patients with BD; and 42.9% for AC, 33.3% for AA, and 23.8% for CC in patients with NBD (Tables 1 and 2). Only the frequency of ApaI A allele in control is higher than that in BD (60% versus 38.5%), and the *p* value is 0.014, but the power is not enough to conclude that ApaI A allele is protective in BD in our study.

The distribution of TaqI genotypes was 36.7% for CT, 43.3% for TT, and 20% for CC in controls; 37.8% for CT, 51.4% for TT, and 10.8% for CC in patients with BD; and 47.6% for CT, 38.1% for TT, and 14.3% for CC in patients with NBD (Tables 1 and 2). The allelic and genotype distributions of TaqI polymorphisms were not different (*p* > 0.05).

The distribution of FokI genotypes was 40% for CT, 10% for TT, and 50% for CC in controls; 40.5% for CT, 5.4% for TT, and 54.1% for CC in patients with BD; and 33.3% for CT, 4.8% for TT, and 61.9% for CC in patients with NBD (Tables 1 and 2). The allelic and genotype distributions of FokI polymorphisms were not different (*p* > 0.05).

Comparing the genotype and alleles frequencies of SNPs (TaqI and FokI) in all disease patients (BD + NBD) compared to controls showed no difference (*p* > 0.05) as summarized in Table 3.

## 4. Discussion

In this study we aimed to demonstrate any association or susceptibility between VDR gene polymorphisms and Behçet's disease group consisting of patients with neuro-Behçet's and Behçet's disease.

Many studies investigated polymorphisms of the VDR gene in various diseases like osteoporosis, cancer, type 1 diabetes, psoriasis, and urolithiasis worldwide and also in Turkish population.

There are only three publications exploring the VDR polymorphisms in Behçet's disease. One of those was performed in Iranian Azeri population and the remaining two were performed in Tunisian population. The Iranian study detected that the only significant difference was found for FokI polymorphism in Behçet's disease, and the f allele was also high in those patients [22]. One Tunisian study stated that TaqI and ApaI polymorphisms might be modestly implicated in BD pathogenesis [23]; but the other study suggested that FokI F allele and F/F genotype associate with BD and that especially FokI polymorphism is associated with vascular manifestations [24].

Although studies investigating VDR SNPs in Behçet's disease are very few, many studies focusing on Turkish population were performed in various diseases.

Bone mineral density is one of the hottest investigation topics of VDR gene polymorphisms. In a study by Kurt et al., it was proved that VDR FokI “FF” genotype constituted risk for lower bone mineral density (BMD) in Turkish postmenopausal women [25]. VDR gene polymorphisms, BsmI, FokI, ApaI, and TaqI were not found to influence the bone metabolism in postmenopausal Turkish women and in a similar group with additional type 1 diabetes [26, 27]. A Turkish study performed in 2009 reported that the distribution of VDR ApaI genotype was similar in osteoporotic, osteopenic, and postmenopausal healthy women [28]. Some studies focused on the Turkish immigrants in Germany in cases with or without additional diseases like diabetes. FokI FF genotype was found to be significantly more prevalent among the Turkish women and Ff-genotyped immigrants showed significantly decreased BMD [29]. Also another study investigating a similar immigrant Turkish population showed that FokI Ff was an important factor for the risk detection for osteoporosis in females [30]. A study performed in Polish patients with systemic lupus (SLE) showed no difference in BsmI polymorphism [31].

The SNPs investigated in our study (FokI (rs10735810), TaqI (rs731236), and ApaI (rs7975232)) have been shown to some extent to be associated with different diseases in the Turkish population compared to control subjects.

These alleles of the SNPs in our control group were similar to those of other studies in the Turkish population (Table 4). For example, a study investigating VDR TaqI and BsmI alleles and the genotype frequencies in female breast cancer patients showed similar results to their control group [32]. Another study suggesting that the only VDR FokI ff genotype was significantly increased in meningioma patients (15.9%) compared with controls (2.5%) and carriers of Fok-I ff genotype had a 6.47-fold increased risk for meningioma development also showed similar alleles frequency in the control group [33]. The same SNPs were also studied in Turkish patients with psoriasis and healthy controls. Dayangac-Erden et al. demonstrated that VDR gene TaqI polymorphism is associated with familial psoriasis in Turkish population with similar SNP frequencies in their control group [34]. The roles of VDR polymorphisms were also investigated in urolithiasis and nephrolithiasis. Goknar et al. demonstrated that the BsmI and TaqI VDR genotypes may be candidate genes causing infantile urolithiasis [35]. But another study showed that none of the VDR ApaI, BsmI, and TaqI polymorphisms created any significant risk for urolithiasis [36]. In a study by Cakir et al., only “B” allele was detected to increase the risk of nephrolithiasis 1.5-fold in 98 patients with calcium oxalate kidney stones [37].

Our results showed no association between the VDR polymorphisms including FokI (rs2228570) and TaqI (rs731236) and patients with neuro-Behçet's (*n* = 21) and Behçet's disease (*n* = 37) compared to healthy controls (*n* = 30). Only the frequency of ApaI A allele in control is higher than that in BD (60% versus 38.5%; *p* = 0.014), but this result was not considered significant because of the small sample size in our study. This difference between the mentioned studies and our results may be due to group size and ethnicity.

## 5. Conclusions

In conclusion, there are many associations between VDR polymorphisms and several diseases; but in our study we were not able to show any relationship between VDR SNPs (FokI (rs2228570) (rs10735810) and TaqI (rs731236)) and Behçet's and neuro-Behçet's disease in Turkish population. ApaI (rs7975232) A allele seems to be protective in BD, but, because of the small sample size in our study, we are not able to conclude that there is any relationship between ApaI A allele and BD. However, future studies with larger patients' numbers may show differences between the abovementioned polymorphisms and Behçet's disease, taking in account that there was no significant association between VDR SNPs and BD in Turkish, Japanese, and Chinese populations shown by several GWAS studies performed [38–40].

## Figures and Tables

**Table 1:** Distribution of genotype and alleles frequencies of VDR SNPs in Behçet's disease and control subjects.

	Controls *n* (%)	Behçet's disease	*p* value
ApaI genotype			
AC	10 (33.3)	15 (42.9)	0.431
AA	13 (43.3)	6 (17.1)	0.021
CC	7 (23.4)	14 (40.0)	0.152
HWE			
*χ* ^2^	2.80	0.319	
*p* value	0.0942	0.571	

ApaI alleles			
A	36 (60)	27 (38.5)	
C	24 (40)	43 (61.5)	0.014

TaqI genotype			
CT	11 (36.7)	14 (37.8)	0.921
TT	13 (43.3)	19 (51.4)	0.514
CC	6 (20)	4 (10.8)	0.294
HWE			
*χ* ^2^	1.511	0.329	
*p* value	0.218	0.565	

TaqI alleles			
C	23 (38.3)	22 (29.8)	
T	37 (61.7)	52 (70.2)	0.29

FokI genotype			
CT	12 (40)	15 (40.5)	0.964
TT	15 (50)	20 (54.1)	0.741
CC	3 (10)	2 (5.4)	0.477
HWE			
*χ* ^2^	0.068	0.143	
*p* value	0.794	0.705	

FokI alleles			
T	42 (70)	55 (74.3)	
C	18 (30)	19 (25.7)	0.57

Differences were considered statistically significant at a *p* value < 0.05.

**Table 2:** Distribution of genotype and alleles frequencies of VDR SNPs in neuro-Behçet's disease and control subjects.

	Controls *n* (%)	Neuro-Behçet's	*p* value
ApaI genotype			
AC	10 (33.3)	9 (42.9)	0.489
AA	13 (43.3)	7 (33.3)	0.472
CC	7 (23.4)	5 (23.8)	0.969
HWE			
*χ* ^2^	2.80	0.382	
*p* value	0.0942	0.536	

ApaI alleles			
A	36 (60)	23 (54.8)	
C	24 (40)	19 (45.2)	0.59

TaqI genotype			
CT	11 (36.7)	10 (47.6)	0.434
TT	13 (43.3)	8 (38.1)	0.708
CC	6 (20)	3 (14.3)	0.598
HWE			
*χ* ^2^	1.511	0.0019	
*p* value	0.218	0.96	

TaqI alleles			
C	23 (38.3)	16 (38.1)	
T	37 (61.7)	26 (61.9)	0.98

FokI genotype			
CT	12 (40)	7 (33.3)	0.628
TT	15 (50)	13 (61.9)	0.400
CC	3 (10)	1 (4.8)	0.493
HWE			
*χ* ^2^	0.068	0.0021	
*p* value	0.794	0.96	

FokI alleles			
T	42 (70)	33 (78.6)	
C	18 (30)	9 (21.4)	0.33

Differences were considered statistically significant at a *p* value < 0.05.

**Table 3:** Distribution of genotype and alleles frequencies of VDR SNPs in all patients (Behçet's disease and neuro-Behçet's disease) and control subjects.

	Controls *n* (%)	All patients	*p* value
ApaI genotype			
AC	10 (33.3)	24 (42.9)	0.389
AA	13 (43.3)	13 (23.2)	0.053
CC	7 (23.4)	19 (33.9)	0.308
HWE			
*χ* ^2^	2.80	0.989	
*p* value	0.0942	0.319	

ApaI alleles			
A	36 (60)	50 (44.6)	
C	24 (40)	62 (55.4)	0.05

TaqI genotype			
CT	11 (36.7)	24 (41.4)	0.669
TT	13 (43.3)	27 (46.6)	0.774
CC	6 (20)	7 (12.1)	0.320
HWE			
*χ* ^2^	1.511	0.213	
*p* value	0.218	0.643	

TaqI alleles			
C	23 (38.3)	38 (32.8)	
T	37 (61.7)	78 (67.2)	0.46

FokI genotype			
CT	12 (40)	22 (37.9)	0.85
TT	15 (50)	33 (56.9)	0.538
CC	3 (10)	3 (5.2)	0.394
HWE			
*χ* ^2^	0.068	0.074	
*p* value	0.794	0.785	

FokI alleles			
T	42 (70)	88 (75.8)	
C	18 (30)	28 (24.2)	0.40

Differences were considered statistically significant at a *p* value < 0.05.

**Table 4:** Comparison of our results of alleles frequencies obtained in the control group with other publications performed in Turkish population.

		Our results	Gunes et al. [36] *n* = 150	Dayangac-Erden et al. [34] *n* = 100	Toptaş et al. [33] *n* = 122	Gogas Yavuz et al. [26] *n* = 134	Buyru et al. [32] *n* = 27	Cakir et al. [37] *n* = 70	Kılıç et al. [41] *n* = 96
ApaI	A	0.60	0.37	0.57	—	0.65	—	0.614	0.54
rs7975232	C	0.40	0.63	0.425	—	0.35	—	0.386	0.46
FokI	T	0.70	—	0.73	0.717	0.84	—	0.736	0.68
rs2228570	C	0.30	—	0.27	0.283	0.16	—	0.264	0.32
rs10735810									
TaqI	A	0.62	0.65	0.595	0.731	0.65	0.615	0.65	0.65
rs731236	C	0.38	0.35	0.405	0.273	0.35	0.384	0.35	0.35
Significancy in disease		No significancy in Behçet's and neuro-Behçet's disease	No significancy in urolithiasis	TaqI in psoriasis	No significancy in brain cancer	No significancy in type 1 diabetes	No significancy in breast cancer	No significancy in nephrolithiasis	No significancy in atopic dermatitis
